# Generalized Propensity Score Matching with Multilevel Treatment Options

**DOI:** 10.36469/9847

**Published:** 2013-03-11

**Authors:** Onur Baser

**Affiliations:** 1 STATinMED Research and the The University of Michigan, Ann Arbor, MI, USA

**Keywords:** selection bias, regression analysis, multilevel treatment, propensity score matching

## Abstract

**Background:** Although conventional form of propensity score matching (PSM) is widely used in outcomes research field, its application on multilevel treatment is limited.

**Objectives:** This article reviews PSM and illustrates their use when there are more than two treatment choices, which is very common in health services research.

**Methods:** Generalized PSM technique was applied to commercial claims data to estimate the treatment effect of reliever only, controller only and combination therapy of patients with asthma. The propensity score is estimated using multinomial logistic regression. The outcome variable was total annual health care costs. Inverse probability weighting was applied to calculate risk adjusted costs. Results are compared with multivariate regression analysis, where the generalized linear model is used with gamma family and log link function.

**Results:** Based on the study’s definitions of an asthma episode, we obtained a sample that included 25,124 patients in fee-for-service (FFS) plans and 6,603 patients in non-FFS plans. Under each plan type, patients who were prescribed three different treatment options were significantly different in terms of their demographic and clinical characteristics. Compared to combination therapy under FFS group, the difference of the total health care costs among reliever therapy and controller only group was significant ($728 and $1,216, respectively). Under non-FFS group, reliever only therapy totaled $1,266; controller only therapy was $1,959, and combination therapy totaled $1,933. Although the cost difference between reliever only and combination therapy was significant, there was no evidence that combination therapy cost more than controller only therapy. There were no significant differences in the multi-level propensity score adjusted results and multivariate regression results.

**Conclusion:** This analysis presents the potential value of generalized PSM methods in health services when there are multilevel treatment options.

## INTRODUCTION

Randomized clinical trials (RCTs) provide solid evidence on casual inference. However, as pointed out by Rossi and Freeman,[1] when the treatment is in its early stages, enrollment demand is minimal, patients have ethical qualm about denying treatment to those perceived to be in need, or when time and many are limited randomization may be difficult to apply or maintain. Since RCTs are carried out using selected populations under idealized, controlled conditions, they are likely to be less generalizable to the population of interest. Under these conditions, real-world data analysis would often be the design of choice.

Real-world data is available in the several forms: (a) large sample trials, (b) registries, (c) electronic medical records, (d) chart reviews, or (e) supplements to traditional RCTs.[2] Unlike RCTs, lack of randomization in assigning individuals to either treatment, and control group make the average treatment effect estimation challenging. Average treatment effect estimation in real-world data analysis often requires adjustment for differences in pre-treatment variables because of the possibility of overt bias. When evaluating treatment effects, overt bias exists because the treatment and control groups are different in terms of certain observables factors.

PSM and regression analysis are two statistical techniques to remove overt bias. Generally, regression analysis assumes a set of linear regression between explanatory variables and the outcome of interest. Moreover, if the number of pre-treatment variables is large and shows real differences between the groups of interests, regression analysis is often inadequate.[3] To illustrate, consider the following hypothetical example that adjusts differences in adverse events in a medication dosage comparison. Suppose, the adverse events ranges from 10% to 20% probability per year and the dosage has two values 6mg and 10mg. Under the effect of exposure differences by patient volume, the covariance would adjust the results so that they ostensibly apply to mean value 8mg in each group even though neither group's dosage is at or near this level.

The last decade has seen a broad surge of interest in PSM to estimate average treatment based on real-world data. Several advantages of PSM over regression analysis is outlined in the literature. PSM design is similar to RCTs.[4] In RCTs, patients are randomly assigned to a treatment and control group, and then the outcomes of interest are compared at the end of the trial to calculate average treatment effect. Therefore, "assignment" is done first and "outcome analysis" is done later.

In PSM, there is a similar sequence in the analysis. Outcome variables in the regression analysis, however, are used as a left hand side variable, that is not supposed to be available in the randomization. Therefore, in outcomes research, where investigators from a wide variety of disciplines are involved, such as clinicians, statisticians and econometricians, PSM is a method easily agreed upon. Second, treatment variable is the main exposure variable in estimating average treatment effect, and the PSM focuses directly on the treatment variable.[5] In regression analysis, it is treated the same as the other variables in the explanatory variable set such as age, gender and comorbid conditions. Third, PSM analysis can eliminate non-comparable exposed subjects.[6]

When the explanatory variable distribution overlaps, regression analysis predicts the outcomes variable in treatment group outside of the observed range to form a comparison for the other at common values of the explanatory variables. And the last, when one group has relatively few outcomes compared to the other group, PSM provides robust estimates.[7,8,9]

Current literature on PSM focuses on models using only two potential states, treatment and non-treatment. However, when evaluating certain treatment programs, more complex framework may be necessary since the actual choice set of individuals contains more than just two options. For example, when analyzing more than two treatment options or dosage level, conventional form of PSM is inadequate and extensions are necessary.

Lecner[10] and Imbens[11] outlined a matching methodology that accounts for multilevel treatments. In the next section, we will briefly discuss how conventional propensity scores can be extended to multi-level treatments and provide step-by-step instructions for application. We will then apply the methodology to commercial claims data to estimate the treatment effect among asthma patients with use of controller only, reliever only, and combination therapies.

## METHODS

Empirical methods in health economics have been developed to answer counterfactual questions, such as "what would happen to a patient's health if he or she were subject to an alternative treatment T?" In order to answer this question, we need to find the difference between the patient's outcome with and without the treatment. Let us first define the problem that PSM is trying to solve.

Let $Y_i^T$ be the medical cost of patient *i* in the treatment group, and $Y_i^C$ be the medical cost of patient *i* in the control group. We are interested in the difference $Y_i^T - Y_i^C$, which is the effect of treatment for a given patient. The problem with this calculation, however, is that no single patient will ever be with and without treatment at the same time. Thus, while we do not intend to determine the effect of treatment on a patient in particular, we do intend to learn the average effect that treatment will have on patients: $E[Y_i^T - Y_i^C]$.

In general, researchers have access to data regarding many patients, some of whom have received treatment, while others have not. Thus, the difference between the medical cost of treated patients and the medical cost of patients in the control group is:

$$\begin{aligned} D &= E\left[Y_i^T \middle| Treated\right] - E\left[Y_i^C \middle| Control\right] \\ &= E\left[Y_i^T \middle| T - Y_i^C \middle| C\right] \\ &= E\left[Y_i^T - Y_i^C \middle| T\right] + E\left[Y_i^C \middle| T\right] - E\left[Y_i^C \middle| C\right]. \end{aligned}$$

The first term, $[Y_i^T - Y_i^C \mid T]$ is the *treatment effect* that we intend to isolate. It represents the effect of treatment on the treated: the average difference treatment makes among treated patients.

The PSM technique tries to minimize the difference $E[Y_i^C \mid T] - E[Y_i^C \mid C]$, so that we can estimate the difference of interest, the treatment effect. $E[Y_i^C \mid T] - E[Y_i^C \mid C]$. The selection bias, is due to possible systematic differences between patients who are treated and the others.

A key assumption, when there are only two choices in the PSM method is that potential outcomes are independent of the treatment, conditional on the set of covariates. As a result, with $p(X)$ equal to the probability that $T = 1$ given $X$, $E[Y_i^C \mid T] - E[Y_i^C \mid C] = 0$. Thus, if we estimate the propensity score and then compare observations that have a similar propensity score, we can eliminate observable selection bias and isolate the treatment effect.

Because PSM employs predicted probability of group membership based on observed characteristics, any model producing a consistent probability estimate is appropriate. When the choice set is binary, logit models are the most common model applied to estimate propensity scores. When the choice set has more than two categories, adaption of generalized propensity score implies discrete response models. If the values of the treatment are qualitatively distinct and without logical ordering, such as different drug treatment types and no treatment, then one can use multinomial logit models. If the treatment choices correspond ordered levels of treatment, such as the dose of a drug, ordered logit regressions can be applied.

For multinomial logit regression, assume we have three categories: Treatment A, Treatment B and No Treatment, and explanatory variable set $X$, which usually contains age, gender, comorbid conditions, etc. Thus, our selection variable y contains three values 1, 2 and 3. Note that the values, although can be coded as 1, 2 and 3, they are "unordered" in the sense that category 1 (Treatment A) is not less than category 2 (Treatment B), but is less than category 3 (No treatment). In the multionomial logit model, we will estimate a set of coefficients, $\beta_1$, $\beta_2$, and $\beta_3$ corresponding to each category:

$$\Pr(y = Treatment\ A) = \frac{e^{X\beta_1}}{e^{X\beta_1}+e^{X\beta_2}+e^{X\beta_3}},$$

$$\Pr(y = Treatment\ B) = \frac{e^{X\beta_2}}{e^{X\beta_1}+e^{X\beta_2}+e^{X\beta_3}},$$

$$\Pr(y = No\ Treatment) = \frac{e^{X\beta_3}}{e^{X\beta_1}+e^{X\beta_2}+e^{X\beta_3}}$$

Note that each of these coefficients has a dimension that is equal to number of explanatory variables used in the regression.

In ordered logit models, linear function of explanatory variables and a set of cut-points estimates the underlying score. Let the random error term $u_j$ is assumed to be logically distributed, $\beta_1, \beta_2, \ldots, \beta_k$ are the coefficients, and $\tau_1, \tau_2 \ldots, \tau_{k-1}$ are the possible treatment choices, such as 6mg, 8mg and 10mg. Then by taking $\tau_0$ as - ∞ and $\tau_k$ as ∞, the probability of observing treatment can defined as follows:

$$\Pr(treatment_j = i) = \Pr\ (\tau_{k-1} < \beta_1 x_{1j} + \beta_2 x_{2j} + \cdots + \beta_k x_{kj} + u_j \leq \tau_i\ )$$

After estimating the probabilities (propensity scores) from multinomial logit or ordered logit depending on whether levels of treatment are qualitatively distinct from the case, the final step is to estimate the conditional expectation of outcomes given treatment level. In other words we will estimate the average response at treatment level $t$ as the average conditional expectations averaged over empirical distribution of the treatment variables:

*In STATA these probabilities can be estimated with "predict" command after running "mlogit". In SAS, we need the following set of commands:

```
proc logistic data=xxx;
class y (ref="xx")/param=ref;
model y=x1 x2 x3/link=glogit;
output out=prob predicted=phat;
run;
```

+In STATA, "ologit" command can be used to run the ordered logit regression. Then "predict" commands provide the probabilities. In SAS, we need the following set of commands:

```
proc logistic data=xxx;
class y (ref="xx")/param=ref;
model y=x1 x2 x3/link=logit;
output out=prob predicted=phat;
run;
```

where $I(t)$ is the binary treatment level indicator.

## CASE STUDY

The application is presented to estimate the treatment effects of asthma patients with the use of controller only, reliever only and combination medication. The data used commercial claims files. The following five variables were available in this database: age, gender, International Classification of Disease 9th Revision Clinical Modification (ICD-9-CM) codes, plan type, and geographic region.

Patients were included in the study if they (a) had at least two outpatient claims with a primary or secondary diagnosis of asthma; or (b) had at least one emergency room (ER) claim with a primary diagnosis of asthma, and a transaction for an asthma drug 90 days prior to, or 7 days following, the ER claim; or (c) had at least one inpatient claim with a primary diagnosis of asthma; or (d) had a secondary diagnosis of asthma and a primary diagnosis of respiratory infection in an outpatient or inpatient claim; or (e) had at least one drug transaction for a(n) anti-inflammatory agent, oral antileukotrienes, long-acting bronchodilator, or inhaled or oral short-acting beta-agonistic.

To ensure that individual records were complete and that the analytic sample would be representative of the population of patients of interest, a number of exclusions were imposed. In particular, patients were excluded if they (a) had a diagnosis of chronic obstructive pulmonary disease, emphysema, or chronic bronchitis; (b) were pregnant at some stage during the study period; (c) were not continuously enrolled in a health plan for 24 months; (d) were enrolled in health maintenance organizations (HMOs) and capitated point of service (POS) plans; or (e) were elderly, defined as ages 65 and over.

The dependent variable was total health expenditure, calculated as the sum of inpatient, outpatient, and pharmaceutical expenditures for all medical care services. This included all services paid for by insurance, as well as co-payments and deductibles paid out-of-pocket.

Asthma drugs can be envisioned as being primarily reliever medication or as being primarily controller medication. Therefore, we divided treatment categories into three parts: 1) Controller patients prescribed medication (such as inhaled anti-inflammatory agents, oral corticosteroids, oral anti-leukotrienes, and long-acting bronchodilators) to control pulmonary inflammation and prevent acute asthmatic exacerbation; 2) Reliever patients were prescribed medication to relieve symptoms in an acute asthmatic exacerbation (i.e., drugs categorized as anti-holinergics or inhaled short-acting beta-agonists); and 3) Combination patients were prescribed both controller and reliever medications.

Based on the definitions of asthma episodes discussed above, we obtained a sample that included 25,124 patients in fee-for-service (FFS) plans and 6,603 patients in non-FFS plans. Table 1 presents descriptive statistics of the sample, stratified by FFS and non-FFS plans, and then stratified by treatment type. Patients in FFS plans averaged 34 years of age, compared to 35 years for non-FFS plans; the FFS-plan patients were also more likely to be female. In addition, patients in FFS plans were more likely than patients in non-FFS plans to reside in the North Central U.S. region. Substantial differences in mean income between the FFS and non-FFS plans were evident from county-level U.S. census data linked to claims data. Patients in FFS plans appear to be sicker than those in non-FFS plans. The former have a higher percentage of asthma-specific comorbidities and have a higher number of major diagnostic categories. As expected from these differences, a likelihood test was conducted to examine whether separate models required for FFS and non-FFS samples concluded that we should estimate separate multinomial logistic models for FFS and non-FFS samples to estimate propensity scores.

In terms of treatment types, patients prescribed combination therapy in FFS plans show substantial differences in demographic and clinical factors relative to those prescribed reliever only medication. Patients prescribed reliever-only medication were younger, healthier, more likely to live in the North Central region, more likely to have a lower income, and less likely to live in the South. In contrast, patients who were prescribed controller only medication were more likely to have a higher income level, less likely to live in the North Central region, and more likely to live in the South, relative to those prescribed combination therapy.

Income differences disappeared among the treatment types for patients in non-FFS plans. The remaining trends were similar to the group in the FFS plans. Because of these observed differences in patient characteristics, adjustment is necessary to compare the total health care expenditure for each type of treatment. Findings may be confounded because of these differences.

**Table 1.** Descriptive Tables

| **Fee For Service** | | | | | | | | |
|---|---|---|---|---|---|---|---|---|
| | **Combination Therapies** | | **Rellever Only** | | **P-value** | **Controller Only** | | **p-value** |
| **Number of Observations** | 11427 | | 11049 | | | 2648 | | |
| | **Mean** | **STD/N** | **Mean** | **STD/N** | | **Mean** | **STD/N** | |
| **Age** | 33.97 | 18.25 | 30.50 | 18.07 | 0.0000 | 32.71 | 17.96 | 0.0012 |
| **Female** | 58.62% | 6699 | 57.23% | 6323 | 0.0339 | 58.19% | 1541 | 0.6861 |
| **% Geographic Region** | | | | | | | | |
| North Central | 82.97% | 9481 | 85.13% | 9406 | 0.0000 | 78.78% | 2086 | 0.0000 |
| South | 10.63% | 1215 | 9.19% | 1015 | 0.0003 | 13.10% | 347 | 0.0003 |
| West | 4.55 | 520 | 3.90% | 431 | 0.0155 | 4.72% | 125 | 0.7064 |
| **% County Average Income** | | | | | | | | |
| $15-20K | 16.29% | 1862 | 18.13% | 2003 | 0.0003 | 14.31% | 379 | 0.0120 |
| $20-25K | 38.75% | 4428 | 39.27% | 4339 | 0.4241 | 38.26% | 1013 | 0.6374 |
| $25-35K | 32.62% | 3728 | 32.18% | 3556 | 0.4805 | 32.06% | 849 | 0.5777 |
| >$35K | 11.79% | 1347 | 9.94% | 1098 | 0.0000 | 15.11% | 400 | 0.0000 |
| **% Asthma-specific Comorbidities** | | | | | | | | |
| Allergic Rhinitis | 31.14% | 3558 | 18.37% | 2030 | 0.0000 | 32.25% | 854 | 0.2655 |
| Migrain | 6.69% | 764 | 5.10% | 564 | 0.0000 | 6.04% | 160 | 0.2282 |
| Depression | 11.54% | 1319 | 9.77% | 1079 | 0.0000 | 10.57% | 280 | 0.1569 |
| GI Disorders | 25.55% | 2920 | 21.33% | 2357 | 0.0000 | 24.21% | 641 | 0.1510 |
| Sinusitis | 27.85% | 3182 | 23.20% | 2563 | 0.0000 | 22.62% | 599 | 0.0000 |
| Anxiety | 2.57% | 282 | 2.40% | 265 | 0.7356 | 2.87% | 76 | 0.2362 |
| **# Major Diagnostic Categories** | 6.48 | 2.39 | 6.06 | 2.15 | 0.0000 | 6.32 | 2.15 | 0.0008 |

**Table 1.** Descriptive Tables—Continued

| **Non-Fee For Service** | | | | | | | | |
|---|---|---|---|---|---|---|---|---|
| | **Combination Therapies** | | **Reliever only** | | **p-value** | **Controller only** | | **p-value** |
| **Number of observation** | 2881 | 3176 | | 546 | | | | |
| | **Mean** | **STD/N** | **Mean** | **STD/N** | | **Mean** | **STD/N** | |
| **Age** | 24.61 | 15.97 | 22.79 | 15.65 | 0.0000 | 27.00 | 15.28 | 0.0009 |
| **Female** | 48.59% | 1400 | 50.76% | 1612 | 0.0929 | 50.00% | 273 | 0.5468 |
| **% Geographic Region** | | | | | | | | |
| North Central | 13.26% | 382 | 13.57% | 431 | 0.7227 | 12.27% | 67 | 0.5304 |
| South | 54.43% | 1568 | 49.43% | 1570 | 0.0001 | 44.32% | 242 | 0.0000 |
| West | 14.02% | 404 | 17.19% | 546 | 0.007 | 23.26% | 127 | 0.0000 |
| **% Country Average Income** | | | | | | | | |
| $15-20K | 4.30% | 124 | 4.03% | 128 | 0.5940 | 3.85% | 21 | 0.6260 |
| $20-25K | 18.60% | 536 | 17.07% | 542 | 0.1178 | 19.23% | 105 | 0.7308 |
| $25-35K | 65.26% | 1880 | 65.62% | 2084 | 0.7673 | 61.90% | 338 | 0.1331 |
| >$35K | 10.97% | 316 | 12.66% | 402 | 0.0423 | 14.47% | 79 | 0.0189 |
| **% Asthma-Specific Comorbidities:** | | | | | | | | |
| Allergic Rhinitis | 24.51% | 706 | 10.89% | 346 | 0.0000 | 29.67% | 162 | 0.0109 |
| Migriane | 5.35% | 154 | 3.18% | 101 | 0.0000 | 6.23% | 34 | 0.4068 |
| Depression | 6.04% | 174 | 5.73% | 182 | 0.6095 | 7.51% | 41 | 0.1941 |
| GI Disorders | 18.36% | 529 | 15.99% | 508 | 0.0146 | 20.15% | 110 | 0.3262 |
| Sinusitis | 22.18% | 639 | 16.15% | 513 | 0.0000 | 21.25% | 116 | 0.6291 |
| Anxiety | 1.67% | 48 | 1.64% | 52 | 0.9300 | 2.56% | 14 | 0.1489 |
| **# Major Diagnostic Categories** | 5.05 | 1.68 | 4.81 | 1.57 | 0.0000 | 5.38 | 1.69 | 0.0000 |

The probability of being in each treatment group using a multinomial logit regression was estimated. Coefficients are the log odds of a patient receiving the reliever medication alone, the controller medication alone, or a combination therapy. Overall, both the FFS model and the non-FFS model significantly estimated the variation in the selection. *((Prob> x2)<0.0000).*

For the FFS model, older patients were less likely to be treated with relievers alone or controllers alone, as opposed to combination therapy.

Females were significantly more likely to be prescribed a reliever only treatment rather than a combination treatment. Residents of the Northeast region (reference category) and those living in a county with the highest category of average income had significantly increased odds of receiving combination therapy rather than controller therapy. There were no significant differences between the reliever only and combination only therapies by residential regions or by the county's average income level. The presence of sinusitis was associated with a decreased likelihood of receiving reliever only therapy or controller only therapy relative to combination therapy. Allergic rhinitis reduced the odds of receiving reliever only therapy but did not have a significant impact on controller only therapy. The other comorbidities exerted no significant impact on the choice of drug therapy.

**Table 2.** Multinomial Logit Regression

| | Fee For Service | | | Non-Fee For Service | | |
|---|---|---|---|---|---|---|
| **Reliever Only** | **Coefficient** | **STD** | **P-values** | **Coefficient** | **STD** | **P-values** |
| Age | -0.0105 | 0.0008 | 0.0000 | -0.0090 | 0.0017 | 0.0000 |
| Female | 0.0664 | 0.0285 | 0.0200 | 0.1455 | 0.0537 | 0.0070 |
| North Central | 0.1062 | 0.1024 | 0.3000 | 0.0379 | 0.0953 | 0.6910 |
| South | -0.1032 | 0.1101 | 0.3490 | -0.0595 | 0.0756 | 0.4310 |
| West | -0.0788 | 0.1210 | 0.5150 | 0.2430 | 0.0929 | 0.0090 |
| $15-20K | 0.1407 | 0.1941 | 0.4680 | 0.1976 | 0.3320 | 0.5520 |
| $20-25K | 0.0458 | 0.1924 | 0.8120 | 0.1390 | 0.3123 | 0.6560 |
| $25-35K | 0.0220 | 0.1925 | 0.0909 | 0.2511 | 0.3078 | 0.4150 |
| >$35K | -0.1140 | 0.1958 | 0.5600 | 0.3409 | 0.3186 | 0.2850 |
| Allergic Rhinitis | -0.6717 | 0.0327 | 0.0000 | -0.9187 | 0.0737 | 0.0000 |
| Migraine | -0.1111 | 0.0603 | 0.0650 | -0.3554 | 0.1382 | 0.0100 |
| Depression | -0.0822 | 0.0463 | 0.0760 | 0.0558 | 0.1178 | 0.6360 |
| GI Disorders | -0.0559 | 0.0367 | 0.1280 | -0.0210 | 0.0783 | 0.7880 |
| Sinusitis | -0.1133 | 0.0324 | 0.0000 | -0.2048 | 0.0703 | 0.0040 |
| Anxiety | 0.1376 | 0.0897 | 0.1250 | 0.1333 | 0.2115 | 0.5290 |
| # Major Diagnositc Categories | -0.0316 | 0.0080 | 0.0000 | -0.0297 | 0.0202 | 0.1400 |
| Constant | 0.5676 | 0.2204 | 0.0100 | 0.3441 | 0.3263 | 0.2920 |
| **Controller Only** | **Coefficient** | **STD** | **P-values** | **Coefficient** | **STD** | **P-values** |
| Age | -0.0027 | 0.0013 | 0.0350 | 0.0071 | 0.0030 | 0.0190 |
| Female | 0.0503 | 0.0456 | 0.2700 | -0.0184 | 0.0966 | 0.8490 |
| North Central | -0.5884 | 0.1297 | 0.0000 | -0.1709 | 0.1728 | 0.3230 |
| South | -0.3040 | 0.1411 | 0.0310 | -0.2482 | 0.1362 | 0.0680 |
| West | -0.4782 | 0.1620 | 0.0030 | 0.4316 | 0.1530 | 0.0050 |
| $15-20K | 0.7360 | 0.4047 | 0.0690 | 0.1175 | 0.6587 | 0.8580 |
| $20-25K | 0.8382 | 0.4021 | 0.0370 | 0.2973 | 0.6236 | 0.6330 |
| $25-35K | 0.7850 | 0.4022 | 0.0510 | 0.2528 | 0.6171 | 0.6820 |
| >$35K | 1.0927 | 0.4050 | 0.0070 | 0.4783 | 0.6319 | 0.4490 |
| Allergic Rhinitis | 0.0792 | 0.0476 | 0.0960 | 0.2432 | 0.1075 | 0.0240 |
| Migraine | -0.0320 | 0.0935 | 0.7320 | -0.0861 | 0.2058 | 0.6760 |
| Depression | -0.0624 | 0.0733 | 0.3950 | -0.0289 | 0.1921 | 0.8810 |
| GI Disorders | -0.0032 | 0.0578 | 0.9550 | -0.1433 | 0.1341 | 0.2850 |
| Sinusitis | -0.2772 | 0.0528 | 0.0000 | -0.1587 | 0.1200 | 0.1860 |
| Anxiety | 0.2469 | 0.1342 | 0.0660 | 0.2404 | 0.3182 | 0.4500 |
| # Major Diagnostic Categories | -0.0142 | 0.0126 | 0.2610 | 0.1157 | 0.0335 | 0.0010 |
| Constant | -1.5651 | 0.4257 | 0.0000 | -2.6598 | 0.6454 | 0.0000 |
| **Number of obs** | 25124 | | | | 6603 | |
| **LR chl2(32)** | 1046.56 | | | | 390.08 | |
| **Prob>chi2** | 0.0000 | | | | 0.0000 | |

For patients in non-FFS plans, the effects of age and gender were similar to those in the FFS analysis. Living in the West reduced the odds of being prescribed reliever only therapy or controller only therapy relative to combination therapy. None of the county-level income variables from the census was statistically significant. The presence of allergic rhinitis, migraine, and sinusitis decreased the odds of receiving reliever only medication relative to combination therapy. For patients prescribed controller therapy, the only comorbidity associated with a significant effect was the presence of allergic rhinitis, which increased the odds of receiving controller-only therapy relative to combination therapy. Higher numbers of unique three digit ICD-9-CM codes significantly decreased the odds of receiving a controller therapy relative to combination therapy.

After estimating each model, we calculated the probability of being in each treatment type and used these probabilities as weights to analyze the outcome variables.

In Table 3, outcome estimates for each of the treatment arms for the FFS and non- FFS group are provided.

**Table 3.** Analysis of Outcome Variables

| **Fee for Service** | | | | | |
|---|---|---|---|---|---|
| **Outcome Variable** | **Combination** | **Reliever Only** | | **Controller Only** | |
| Total Health Care Cost | Mean | Mean | p-value | Mean | p-value |
| Unadjusted | $4263 | 2792 | 0.0000 | 2965 | 0.0000 |
| Propensity Score Adjusted | $4039 | 3311 | 0.0000 | $2823 | 0.0000 |
| Multivariate Adjusted | $2587 | 1826 | 0.0000 | $1907 | 0.0000 |
| **Non-Fee for Service** | | | | | |
| **Outcome Variable** | **Combination** | **Reliever only** | | **Controller Only** | |
| Total Health Care Cost | Mean | Mean | | Mean | |
| Unadjusted | $2031 | 1285 | 0.0000 | $2371 | 0.3944 |
| Propensity Score Adjusted | $1933 | 1266 | 0.0000 | $1959 | 0.9285 |
| Multivariate Adjusted | $1265 | 841 | 0.0000 | $1161 | 0.4040 |

The first row presents the unadjusted mean for total health care costs. The difference of total health care cost between reliever therapy and combination therapy was $1,471 for the FFS group and $746 for the non-FFS group. The estimates were $1,298 for the FFS group, and a savings of $340 for the non-FFS group between controller only and combination therapy. All of these differences were confounded with patients' demographic and clinical characteristics.

The second row presents propensity score adjusted estimates. The difference in the total health care costs between reliever and combination therapy for the FFS group was $728 - a statistically significant difference. As expected, the difference was smaller than the unadjusted mean, because patients in the reliever group were younger and healthier; therefore, the unadjusted mean for this comparison reflected an upward bias. The propensity score-adjusted difference between patients prescribed controller only medication and combination medication was $1,216. This adjusted difference represented a difference of only $82 from the unadjusted mean, because these groups of people were similar before PSM. Therefore, we would anticipate little adjustment in price after controlling for confounding factors. We can see similar trends in the non-FFS group. Reliever only therapy totaled $1,266, controller only therapy was $1,959, and combination therapy totaled $1,933. Although the cost difference between reliever only and combination therapy was significant, there was no evidence that combination therapy cost more than controller only therapy.

We also adjusted the heterogeneity in the sample by using multivariate analysis. Multivariate analysis and PSM control for the observed differences in treatment groups. Therefore multivariate analysis serves as a sensitivity analysis in our application. We modeled health care expenditures as a function of patients' demographic and clinical factors used in the multinomial logit, and we added two dummy variables: one for reliever only and one for controller only. Following the principles proposed by Manning and Mullahy,[12] we used a generalized linear model with a log-link function and gamma family. Marginal effects from estimated parameters are presented in the last row of Table 3. The differences in total health care expenditure by each of the three treatment arms were similar to the ones we see in propensity score-adjusted differences. For the FFS group, comparing combination therapy with reliever only therapy, the difference was $761, according to multivariate analysis ($728 when compared to PSM).

The expenditure difference between controller-only therapy and combination therapy was $1,280 ($1,266 in PSM). For the non-FFS group, the estimated cost of combination therapy was $1,265, reliever only therapy was $841, and controller only therapy was $1,161. The differences in cost estimates according to multivariate and propensity score adjustment were not statistically significant.

## DISCUSSION

In many circumstances, the best source of information on estimating average treatment effect involves retrospective analysis from the real-world data. PSM is a great tool for estimating average treatment effect by providing a design similar to clinical trials and adjusting for confounding factors.

The conventional form of matching is widely used in health services research. Statistical properties have been investigated by many researchers and guidelines have been provided in order to help choose among the different types of PSM techniques.[13] The discussion in this article for PSM does not give detailed or rigorous treatment of theory that underlines the PSM technique. The author encourages curious readers to consult series of articles by Rubin[4,14-18] on the conventional form of PSM and Imben's article on multi-level propensity matching.[11]

The extension of the conventional form of matching technique to the multilevel treatment essentially involves a weighting scheme. Weights are determined by the inverse of estimated propensity scores and propensity scores are estimated using multinomial or ordered logit models. Inverse probability estimation is frequently used in outcomes research when estimating costs from censored data.[19,20]

Generalized propensity score models as in conventional forms rests on a critical assumption of "strong ignorability".[21] When one applies PSM, it is implicitly assumed that the choice of treatments is not based on the benefits of alternative treatments once it is conditioned to the set of explanatory variable set. In our example, after controlling for baseline characteristics, the physician chooses among reliever only, controller only or combination therapy randomly. This assumption may not be true for every treatment or on the range of covariates involved in the analysis. Therefore, a caution is necessary.

Selection of covariates is an important step in the multinomial or ordered logit regression when estimating the propensity score. The causality relationship among covariates, outcomes, and treatment variables should be derived from theoretical relationships and sound knowledge of previous research. Because including variables only weakly related to treatment assignment usually reduces bias more than it increases variance-using matching, under most conditions these variables should be included. Interaction terms should be tested, and to avoid overmatching, one should not include any variables that are measured during the treatment.[13,22]

The estimation power of the models and significance of the joint effect variables would provide strong evidence if matching produces balanced groups. F-statistics for the significance of joint effect or generalized form of receive operator curve to detect classification power can be used.[23]

One final point is to consider before applying generalized PSM is identifying substantial overlaps among the groups. Every inclusion/exclusion criteria should be applied to all groups. If there is a lack of overlap, one can use a method that provides a systematic approach to account for subpopulations with limited overlap in the explanatory variable set.[24] In particular, method balances two opposing effects (a) the increase in variance of the estimated average treatment effect due to smaller (subpopulation) sample size, and (b) the decrease in variance of the estimated average treatment effect due to discarding sensible observations whose efficient comparable representative is missing. Then estimates optimal subpopulation average treatment effect.

In our analysis we compared the propensity score matching and multivariate regression analysis since both of these techniques are designed to remove observed bias in real world data analysis. We found that the results were not significantly different from each other. Recent systematic literature reviews compared the estimates of relationship between exposures with those obtained multivariate models.[25] Statistical significance differed between the two methods in only 10% of cases. In other study, it has been showed that propensity score and regression model approaches greater than 20% only in 13% of cases.[26]

It is important to note that neither propensity score matching nor regression analysis addresses or resolves problems due to imbalances in unmeasured factors. When unobservable bias exists, there are more advanced techniques such as bounding approach[27] difference-in-difference estimators[28] or instrumental variable approach exists.[29,30] However these estimations are also confounded by their own limitations. For example, the bounding approach does not provide point estimation rather provides a range of estimators. Difference-in-difference estimators are highlyfor functional misspecification. Instrumental variable approaches can provide results that are more biased than PSM analysis when the instruments are weak.

## CONCLUSION

Causal inference is challenging when studying real world data because of the inevitable self-selection. PSM addresses selection bias issue due to observable factors. However, its conventional form matches only two groups. Under many circumstances the choice sets include more than two groups and generalization of the technique increases the applicability of the matching algorithm. We showed how one can apply the matching when there is multilevel treatment option. The method can be easily applied using standard statistical software.

## CONFLICT OF INTEREST DECLARATION

The author declares that there are no competing interests.
